# Supervised machine learning-based bias risk of prognostic models for total knee or hip arthroplasty patients: A systematic review

**DOI:** 10.1097/MD.0000000000045230

**Published:** 2025-10-17

**Authors:** Hongxia Zhang, Lina Jiang, Jiang Zheng, Chuanbo Li

**Affiliations:** a Department of Orthopedics, Banan Hospital Affiliated to Chongqing Medical University, Chongqing, China; b Leshan City Vocational and Technical College Nursing Department, Leshan, China.

**Keywords:** arthroplasty, prognostic model, risk bias, supervised machine learning, systematic review

## Abstract

**Background:**

As various machine learning (ML) algorithms have become more popular in orthopedic surgery, the research quality of these models requires further evaluation, and the methodological quality of the models still needs to be clarified. This study aimed to comprehensively analyze and evaluate the potential bias and applicability of existing studies of supervised ML-driven prognostic risk prediction models focusing on total knee arthroplasty/total hip arthroplasty individuals.

**Methods:**

The China National Knowledge Infrastructure, Wanfang, PubMed, Web of Science, Cochrane Library, and Embase databases were searched from inception to January 20, 2024. The following data were extracted from the selected studies: author, year, joints, sample size, data source, study design, ML methods, ML performance, and primary outcome. The PROBAST checklist was applied to evaluate the risk of bias and applicability in prediction model studies. The protocol is registered in the PROSPERO database (CRD42024501747).

**Results:**

A total of 2909 indexed records were obtained and 32 studies were included, 30 of which had models for internal validation only, 1 study for development and validation, and 1 study for external validation only. The PROBAST evaluation results showed that 1 externally validated model and 29/31 (93%) development models were rated as having a high risk of bias. There was a high risk of bias in the participant and analysis domains.

**Conclusion:**

Almost all supervised ML models have the potential for a high bias risk. Factors contributing to a high bias risk include inadequate sample size, missing data during recruitment, model overfitting, and limited external validation. Adhering to strict standards and implementing comprehensive improvements when constructing prognosis models using supervised ML is crucial.

## 1. Introduction

As the field of clinical nursing progresses towards personalized monitoring, decision-making, and treatment, it is critical to gather data on individual risk profiles.^[1–3]^ Prognostic models, which are complex multivariable risk prediction tools, utilize a range of predictive factors to estimate the probability of a future event occurring within a specific timeframe.^[4]^ These models play a pivotal role in guiding clinical decisions, evaluating care effectiveness, and enhancing patient outcomes.^[5]^ The ongoing development of artificial intelligence (AI) technology offers promising avenues for improving individualized care and catalyzing innovation in medical research.^[6]^ Prognostic models have been widely adopted in various medical fields and settings.^[7–9]^

Machine learning (ML), a subset of AI, employs algorithms to detect patterns in large datasets, enabling machines to learn and make independent decisions or recommendations.^[10,11]^ ML can be categorized into supervised and unsupervised learning. Supervised learning, in particular, uses labeled datasets to train models to predict outcomes in new data, often showing equal or superior predictive power compared to traditional medical research models.^[12]^ In orthopedic surgery, several ML algorithms have gained traction, demonstrating high performance and accuracy in tasks such as fracture image recognition, diagnostic classification, clinical decision-making, and perioperative management.^[13–16]^ A systematic review has shown that ML-based predictive models can forecast a range of orthopedic outcomes, including patient survival, discharge status, and postoperative complications.^[17]^ Nonetheless, challenges remain in the further development and clinical implementation of ML technologies.

In systematic reviews, it is crucial to evaluate the quality of the included studies. The prediction model risk of bias assessment tool (PROBAST) is designed to assess the risk of bias (ROB) and the applicability of diagnostic and prognostic prediction models, whether they are in their development, validation, or update stages, and provides thorough methodological quality assessments.^[18]^ PROBAST highlights how flaws in the design, execution, or analysis of predictive models can lead to a high risk of bias, potentially compromising the precision of the models’ performance predictions.^[19]^ Such inaccuracies could adversely affect clinical decision-making and patient care effectiveness, thus reducing the practical value of the models. Conducting a meticulous quality assessment of predictive model research is therefore vital to ensure the construction of accurate models and to improve their clinical applicability.

In this systematic review, we evaluated the risk of bias and the applicability of supervised ML-based models for predicting the prognosis of patients undergoing total knee arthroplasty (TKA) and total hip arthroplasty (THA). Our aim is to provide insights that may inform future model refinements and clinical practice.

## 2. Methods

In reporting this systematic review, we adhered to the preferred reporting items for systematic review and meta-analysis (PRISMA) statement.^[20]^ The protocol was registered on the PROSPERO platform (CRD42024501747).

### 2.1. Search strategy

The literature search for this study was thorough, utilizing a range of databases: China National Knowledge Infrastructure, Wanfang, PubMed, Web of Science, The Cochrane Library, and Embase. Searches were conducted from the inception of each database through January 12, 2024. Keywords included “ML,” “supervised ML,” “arthroplasty, replacement, Knee,” “arthroplasty, replacement, hip,” “risk prediction model,” “model,” “predictor,” and “Score.” A complete search strategy can be found in Supplementary File 1 (Supplemental Digital Content, https://links.lww.com/MD/Q360). Additionally, a manual examination of the reference lists from selected articles and reviews identified further relevant studies.

### 2.2. Inclusion and exclusion criteria

We constructed the data extraction table based on the critical appraisal and data extraction for systematic reviews of prediction modeling studies (CHARMS) guidelines.^[21]^ Our study is confined to Chinese and English literature, reflecting the population distribution and linguistic predominance (Table 1).

**Table 1:** Selection criteria for the study.

	Inclusion criteria	Exclusion criteria
Participants	Human participants are adults undergoing primary TKA/THA.	1. No human participants. 2. Studies focused on anatomic joints other than the hip and knee. 3. Studies did not include any joint replacement procedures.
Intervention	Developing or validating multivariate predictive models for personalized predictions using supervised machine learning.	1. Research based on unsupervised ML technology. 2. Studies used only linear or logistic regression. 3. The model included only genetic markers or biomarkers as individual predictors. 4. Studies aimed to enhance the image or signal reading.
Comparator (if presented)	Previously validated models.	No competing model.
Study type	Randomized controlled trials, cohort studies, case-control studies.	Review articles, methodological articles, conference abstracts, commentaries, letters, editorials, and publications for which full text was unavailable.
Outcome	Articles assessing prognosis and postoperative outcomes.	NA

NA: not applicable.

### 2.3. Screening process

After retrieval, 2 authors independently screened the studies and refined the selection. The initial phase involved eliminating duplicate studies in accordance with the predetermined selection criteria. Subsequently, the titles and abstracts were carefully examined, followed by a detailed full-text review. Moreover, a systematic review of the references within these studies was carried out to uncover additional relevant research that fulfilled the established criteria. In instances of disagreement regarding study selection, discussions with a third author were held to achieve consensus.

### 2.4. Data extraction

Two independent assessors employed the CHARMS checklist to collect data, and a third party was involved in discussions to reconcile any discrepancies, thereby guaranteeing the accuracy and uniformity of the data collection process. Essential elements covered include authorship, year of publication, affiliations, sample size, data sources, research framework, ML techniques, performance metrics, key results, and other intricate details.

### 2.5. Quality assessment

The current version of PROBAST was employed to assess the methodological quality, risk of bias, and appropriateness of the prognostic model. PROBAST consists of 4 domains – participants, predictors, outcomes, and Analysis – which include 20 signal questions designed to assist in the assessment of risk as low, high, or uncertain. Responses to each signal question are indicated as yes (Y), probably yes (PY), probably no, no (N), or no information. The first 3 domains were appraised in a similar manner, considering their relevance, which is also categorized as low, high, or indeterminate (Table 2).

**Table 2:** PROBAST risk rating description.

Evaluation criterion	Overall risk of bias	Overall applicability	Level of risk
The evaluation results of all signaling questions are “Yes/Probably Yes.”	All domains were rated low risk of bias.	Low applicability risk across all domains in evaluation.	Low
The evaluation result of ≥1 signaling question is “Not/Possibly not. “	≥1 domain is judged to be at high risk of bias.	High applicability risk identified in ≥1 domain.	High
≥1 signaling question evaluation result is “No Information “; the evaluation results of other signaling questions are all “Yes/Probably Yes.”	≥1 domain has unclear bias risk but is low risk in all other domains.	≥1 domain has unclear evaluation results, while other domains have low bias risks.	Unclear

For precision, 2 independent reviewers conducted assessments for each model across all domains in the studies. Discrepancies were resolved through discussion among the authors to achieve consensus. In cases where no explicit recommendation exists, we will opt for the model that demonstrates the greatest discriminative capacity.

Since this research does not involve clinical trials, ethical approval is not required.

### 2.6. Data analysis

The prognostic models in the studies were categorized into 3 types: development, validation, and combination. Different signaling questions suit different types of prediction model evaluations. The PROBAST tool is not applicable if the evaluation pertains to any of these categories. We have excluded signaling questions relevant solely to regression studies, for instance, question 4.9. The findings are presented as percentages, accompanied by corresponding visual representations.

## 3. Results

### 3.1. Study selection

**Figure 1.**
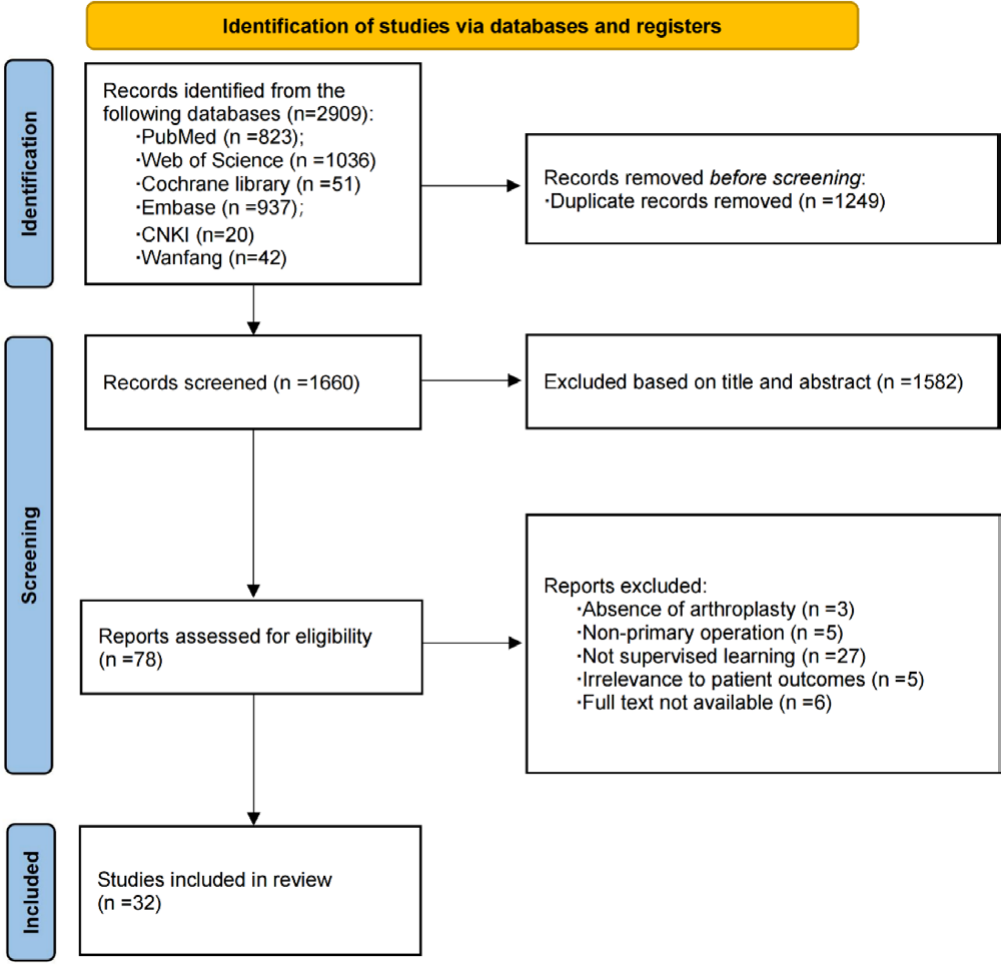
Preferred reporting Items for systematic reviews and meta-analyses (PRISMA) flowchart of the included studies.

The initial query resulted in 2909 records. After the removal of 1249 duplicates, we assessed the eligibility of 1660 titles and abstracts. Subsequent detailed scrutiny led to the exclusion of 27 studies due to their lack of relevance to supervised ML. Furthermore, 3 studies were excluded because they did not pertain to joint replacement, 5 studies were unrelated to primary surgery, 5 studies failed to address the research questions, and 6 studies were inaccessible in full text. In the end, the review incorporated 32 studies and 25 distinct models (Fig. 1).

### 3.2. Study characteristics

The study ultimately included 32 studies conducted between 2015 and 2023, categorized by research objectives. Specifically, 30 studies focused on model development with internal validation,^[22–51]^ one on external validation only,^[52]^ and one on both development and validation.^[53]^ Eight studies targeted THA,^[22,23,27,33,34,36,41,51]^ 16 on TKA,^[24–26,28,30–32,35,40,42,43,45,47,52,53]^ and 8 on a hybrid approach incorporating both procedures.^[29,37–39,44,46,49,50]^ Sample sizes varied significantly, ranging from 100 to 424,354 individuals. Data sources included 18 single-center studies,^[22–26,28,32,33,35,36,38,41,42,45,47–50]^ 3 multi-institutional studies,^[29,44,52]^ ten utilizing public databases,^[27,30,31,34,37,39,40,43,46,51]^ and one combining a single-center study with a public database for internal and external validation.^[53]^ Of the studies, 17 were prospective,^[22,24,27,30–33,35,37,38,43,47–52]^ 14 were retrospective,^[23,25,26,28,34,36,39–42,44–46,53]^ and one was a nested case-control study^[29]^ (Table 3).

**Table 3:** Characteristics of included studies.

Author/Year	Joints	Sample size	Data source	Study design	ML methods	ML Performance^a^	Primary outcome
Development model							
Lungu^[22]^ (2015)	THA	265	Single institution	Prospective cohort study	PA	Sensitivity: 75.0% Specificity: 77.8%	1–2 yr prognosis
Gabriel^[23]^ (2019)	THA	960	Single institution	Retrospective study	RR, Lasso, RF	AUC: 0.761 (0.703–0.820)	LOS
Katakam^[24]^ (2020)	TKA	12,542	Single institution	Prospective cohort study	SGB, RF, SVM, NN, ENP	AUC: 0.760	Prolonged postoperative opioid prescriptions
Chen^[25]^ (2021)	TKA	634	Single institution	Retrospective study	SVM, RF, XGBoost	AUC: 0.888	Transfusion
Chen^[26]^ (2021)	TKA	777	Single institution	Retrospective study	MARS, KNN, SVM, RF, XGBoost, ANN	AUC: 0.903	LOS
Fassihi^[27]^ (2021)	THA	77,145	ACS-NSQIP	Prospective cohort study	ANN	AUC: 0.800	30-d mortality
Han^[28]^ (2021)	TKA	1298	Single institution	Retrospective study	ADBoost, ANN, KNN, DT, BDT, RF, XGBoost	AUC: 0.766 (0.714–0.817)	LOS
Huang^[29]^ (2021)	THA, TKA	12,642	Multiple institution	Nested case-control study	LSTM, RF, DT, KNN, SVM, naïve Bayes classifier	AUC: 0.830 (0.810–0.850)	Transfusion
Jamshidi^[30]^ (2021)	TKA	7589	OAI	Prospective cohort study	DeepSurv, RF, linear/kernel SVM, linear/neural MTLR	AUC: 0.870	Timing and risk of operation
Wei^[31]^ (2021)	TKA	28,742	ACS-NSQIP	Prospective cohort study	ANN	AUC: 0.801	LOS
Yeo^[32]^ (2021)	TKA	10,021	Single institution	Prospective cohort study	ANN, SGB, SVM, RF, ENP	AUC: 0.840	Surgical site infection
Zhang^[33]^ (2021)	THA	1508	Single institution	Prospective cohort study	RF, XGBoost, SVM, Lasso	AUC: 0.760	Patient satisfaction
Zhong^[34]^ (2021)	THA	63,859	ACS-NSQIP	Retrospective study	multivariable logistic regression, ANN, RF	AUC: 0.804	LOS
Chen^[35]^ (2022)	TKA	100	Single institution	Prospective cohort study	XGBoost	AUC: 0.832 (0.748–0.916)	DVT
Cohen-Levy^[36]^ (2022)	THA	7265	Single institution	Retrospective study	ANN, SGB, SVM, ENP	AUC: 0.830 (0.810–0.850)	Transfusion
Rasouli^[37]^ (2022)	THA, TKA	392,661	Cerner health facts	Prospective cohort study	RF, GBT, TE, FCDNN	AUC: 0.873	Venous thromboembolism
Kunze^[38]^ (2022)	THA, TKA	30,703	Single institution	Prospective cohort study	SGB, XGBoost, RF, NN, SVM	C-statistic: 0.750	Hyponatremia
Lopez^[39]^ (2022)	THA, TKA	437,784	ACS-NSQIP	Retrospective study	ANN, BDT	AUC: 0.814	LOS
Mohammed^[40]^ (2022)	TKA	636,062	ACS-NSQIP	Retrospective study	LR, GBT, RF, ANN	AUC: 0.790–0.870	Complications and transfusion
Zheng^[41]^ (2022)	THA	182	Single institution	Retrospective study	RF	AUC: 0.986	6-mo prognosis
Cavazos^[42]^ (2023)	TKA	2093	Single institution	Retrospective study	MPNN	AUC: 0.894	Transfusion
Chen^[43]^ (2023)	TKA	267,966	ACS-NSQIP	Prospective cohort study	ANN, RF, HGB, KNN	AUC: 0.820	LOS
Crawford^[44]^ (2023)	THA, TKA	158	Multiple institution	Retrospective study	SGB, RF, SVM, NN, ENP	AUC: 0.830	Surgical indication
Ding^[45]^ (2023)	TKA	1481	Single institution	Retrospective study	LR, XGBoost, ADBoost, GBT, KNN	AUC: 0.982	DVT, pulmonary embolism
Jia^[46]^ (2023)	THA, TKA	5523	UCSD health	Retrospective study	LR, SVM, balanced random forest, XGBoost, LightGBM	AUC: 0.734	same-day discharge after joint arthroplasty
Klemt^[47]^ (2023)	TKA	10,021	Single institution	Prospective cohort study	ANN, SVM, KNN, ENP	AUC: 0.820	90-d unplanned readmissions
Nam^[48]^ (2023)	TKA	381	Single institution	Prospective cohort study	LR, XGBoost, SVM, RF, MLP, KNN	AUC: 0.782	Patient satisfaction
Park^[49]^ (2023)	THA, TKA	1401	Single institution	Prospective cohort study	RF, SVM	AUC: 0.744–0.749	LOS
Wang^[50]^ (2023)	THA, TKA	6897	Single institution	Prospective cohort study	XGBoost, RF, SVM, LR, BPNN, ensemble	AUC: 0.921 (0.896, 0.936)	DVT
Yeramosu^[51]^ (2023)	THA	19,840	ACS-NSQIP	Prospective cohort study	SGB, RF, SVM, ANN	AUC: 0.831	Non-home discharge
Validation model							
Twiggs^[53]^ (2019)	TKA	330	Multiple Institution	Prospective cohort study	BBN	No Information	Risk of poor prognosis
Development and validation model							
Chen^[52]^ (2023)	TKA	424,354/ 10,196	ACS-NSQIP/ Single institution	Retrospective study	ANN, RF, HGB, KNN	AUC: 0.830–0.840	Non-home discharge

**Figure 2.**
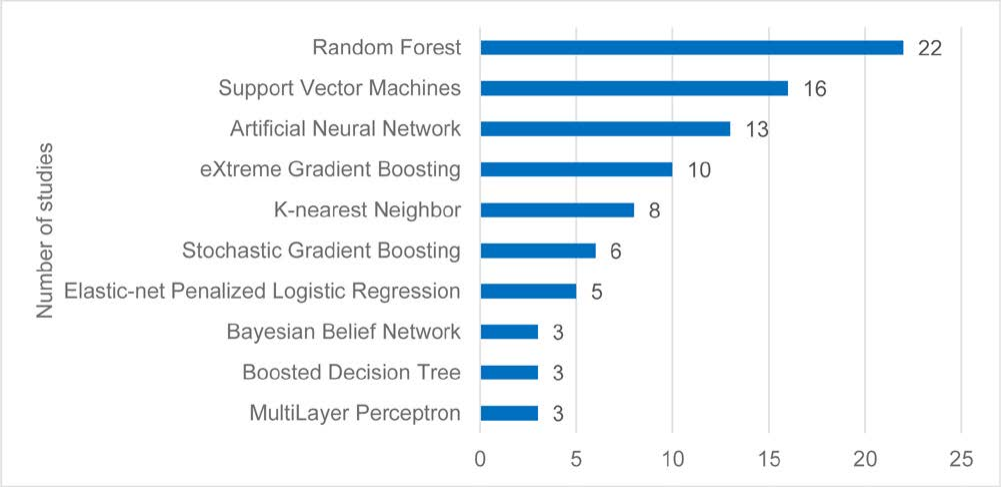
Top 10 machine learning methods for usage.

Fifteen supervised ML algorithms were employed across the studies, involving 25 distinct algorithms, with the top 10 being the most frequently used. The random forest algorithm was the most common (used in 22 studies), followed by support vector machines (16 studies) and artificial neural networks (13 studies). Model performance varied, with one study lacking performance data,^[52]^ one reporting sensitivity and specificity (75.0% and 77.8%),^[22]^ and another a C-statistic of 0.75.^[38]^ Area under the curve ranges were reported in 29 studies (0.835 [0.734–0.936]).^[23–37,39–51,53]^ The most commonly reported primary outcomes were length of stay (8/32 studies, 25%), blood transfusion (5/32 studies, 15.63%), and complications (5/32 studies, 15.63%) (Fig. 2 and Table 3).

### 3.3. Results of the methodological quality assessment

Out of the 31 developed models, 30 were assessed as having a high risk of prognostic model bias, with one model presenting an unclear risk level. Moreover, among these models, 16 development models and one external validation model raised significant concerns regarding their overall applicability. Conversely, 11 development models were deemed to have a low level of concern, while 4 models exhibited unclear applicability ratings. Detailed evaluations for each signal question are provided in Supplementary File 2 (Supplemental Digital Content, https://links.lww.com/MD/Q360).

### 3.4. Participants

**Figure 3.**
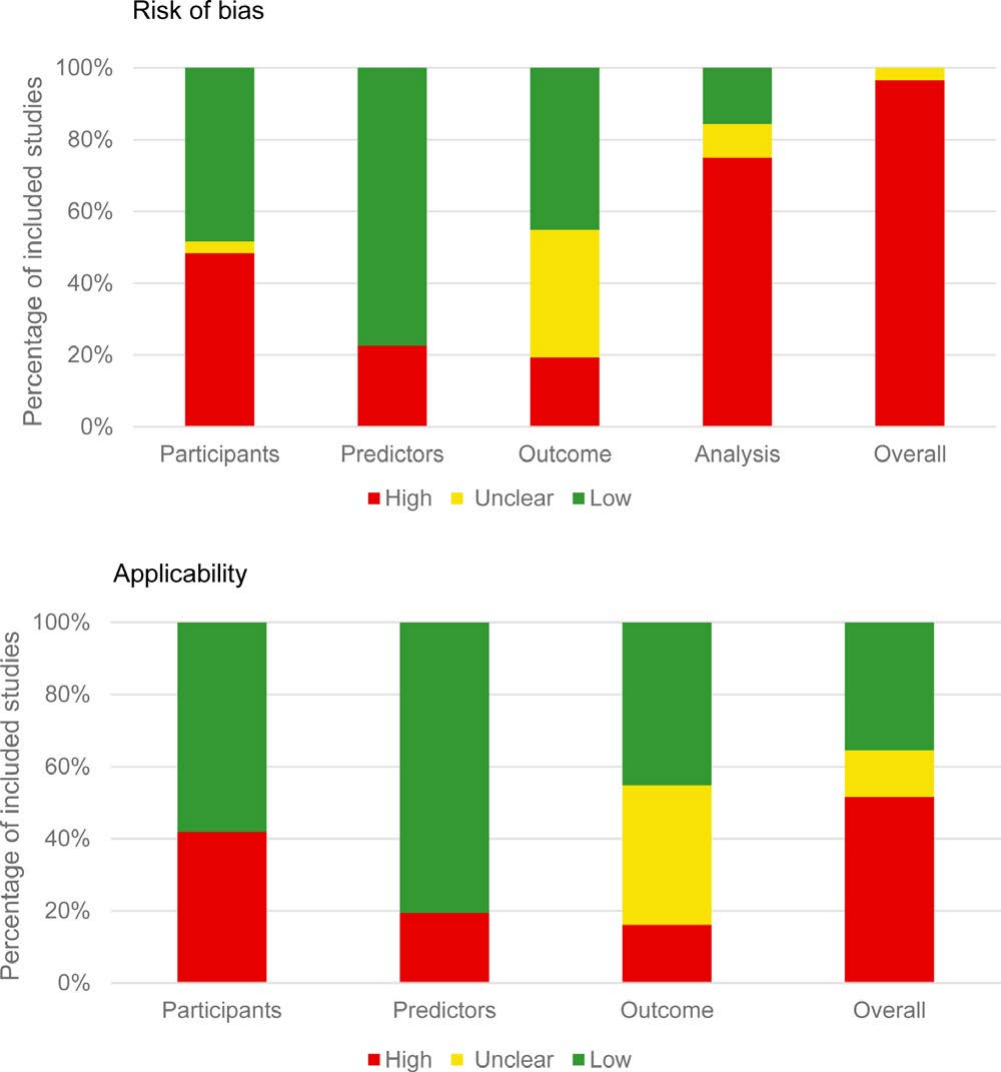
Risk of bias and applicability of the included studies.

Of the 31 developed models, 15 (48%) were rated as having a high risk of bias due to external validation issues in the participant domain (as shown in Fig. 3). Models for 13 (42%) of the participants were derived from available data or retrospective studies, which elevated their bias risk. Furthermore, one model (3%) was developed by a participant using unclear inclusion and exclusion criteria or recruitment strategies, compromising its representativeness of the intended target population (Table 4).

**Table 4:** PROBAST signaling questions for model development in 31 included studies.

	Signaling questions	Risk of bias % (n)		
		Y/PY	N/PN	NI
DOMAIN 1: Participants				
1.1	Data sources	58 (18)	42 (13)	0
1.2	Inclusions and exclusions	90 (28)	3 (1)	7 (2)
DOMAIN 2: Predictors				
2.1	Definition and evaluation of predictors	87 (27)	13 (4)	0
2.2	Blind method	94 (29)	3 (1)	3 (1)
2.3	Availability of all predictors	90 (28)	7 (2)	3 (1)
DOMAIN 3: Outcome				
3.1	Definition and evaluation of outcome	74 (23)	10 (3)	16 (5)
3.2	Prespecified or standard outcome	77 (24)	0	23 (7)
3.3	Exclusion of predictor	70 (22)	7 (2)	23 (7)
3.4	Definition and determination of results	84 (26)	16 (5)	0
3.5	Blind method	80 (25)	0	20 (6)
3.6	Time interval	70 (22)	0	30 (9)
DOMAIN 4: Analysis				
4.1	Number of recruiters	61 (19)	32 (10)	7 (2)
4.2	Treatment of continuous prediction and categorical prediction	32 (10)	16 (5)	52 (16)
4.3	Participant integrity	55 (17)	35 (11)	10 (3)
4.4	Processing of missing data	70 (22)	7 (2)	23 (7)
4.5	Selection of predictors	67 (21)	10 (3)	23 (7)
4.6	Data complexity	84 (26)	3 (1)	13 (4)
4.7	Model performance index	55 (17)	45 (14)	0
4.8	Overfitting and optimism of the model	97 (30)	0	3 (1)

N = no, NI = no information, PN = probably no, PROBAST = prediction risk of bias assessment tool, PY = probably yes, Y = yes.

### 3.5. Predictors

Out of the 31 developed models, 8 (26%) exhibited a high risk of bias within the prediction domain (Fig. 3). Four of these models (13%) utilized diverse definitions and assessment methods for predictors across different participants. The absence of blinding in one model (3%) could potentially elevate the risk associated with the predictor evaluation process. Furthermore, not all candidate predictors demonstrated statistical significance. Additionally, missing data in 2 of the developed models (7%) may impinge upon the predictive model’s applicability in practical scenarios (Table 4).

#### 3.5.1. Outcome

In this study, 6 out of 31 (20%) developed models were assessed as having a high risk of bias, while 11 out of 31 (35%) models, along with one externally validated model, were categorized as having an unclear risk of bias within the externally validated outcome domain (Fig. 3). Furthermore, 3 out of 31 (10%) models failed to employ the appropriate classification for defining outcomes. Despite 24 out of 31 (77%) models and one external validation utilizing uniform, pre-established criteria for outcome determination, 5 out of 31 (16%) models lacked consistency in the threshold categories applied to the measurement of participant outcomes. The results also indicated that 22 out of 31 (70%) development models and one external validation omitted potential predictors. Additionally, the precision of predictor information could have been improved in 25 out of 31 (80%) models and one external validation study when determining outcomes. A total of 22 out of 31 (70%) models demonstrated a reasonable interval between the assessment of predictors and the determination of outcomes, which was sufficient to accurately record outcome events and sample size (Table 4).

#### 3.5.2. Analysis

In the analysis domain, a significant proportion (24 out of 31, or 77%) of the developed models exhibited a high risk of bias. For 10 out of 31 models (32%), the event-per-variable (EPV) ratio was inadequate (EPV < 10), suggesting potential overfitting. Furthermore, over half (16 out of 31, or 52%) of the model development studies failed to adequately report how they handled continuous or categorical variables. Regarding missing data, 11 out of 31 models (35%) excluded cases with missing information without justification, and 7 out of 31 (23%) did not clearly describe their methods for managing such data. A majority (22 out of 31, or 71%) of the models were developed through the comparison and appropriate adjustment of candidate predictors without relying solely on univariate analysis for the selection of predictors. In terms of explaining data complexity, the vast majority (26 out of 31, or 84%) of model developments, along with one external validation, provided a reasonable explanation. While nearly half (14 out of 31, or 45%) of the developed models reported on model discrimination, there was a notable lack of calibration charts or tables to comprehensively assess the models’ predictive performance. Almost all (30 out of 31, or 97%) models utilized appropriate internal validation methods and assessed the impact of any subsequent adjustments on the models’ performance.

## 4. Discussion

The existing AI research has primarily focused on predicting treatment outcomes, with less emphasis on patient outcomes and support for clinical decision-making.^[6]^ ML algorithms are capable of processing complex data to create predictive models that improve patient prognosis and overall health outcomes. However, it is important to recognize that inaccurate predictions by these algorithms in clinical environments could lead to suboptimal patient outcomes.^[54–56]^ Therefore, rigorous assessment and refinement of these models are crucial before they are integrated into routine medical practice.^[57,58]^ Given the current evidence, documenting the effectiveness of such prognostic models for patients undergoing TKA/THA is imperative. In this study, we used supervised ML to evaluate the methodological quality of prognostic models for patients receiving TKA/THA.

The study indicated that the prognostic models under review were significantly susceptible to bias, varied in their applicability, and generally lacked external validation. The domains of participant characteristics and statistical analysis were frequently flagged for high bias risk. The high bias risk in the participant domain primarily stemmed from reliance on existing data or retrospective cohort studies, which can introduce selection bias. The statistical analysis domain exhibited the highest bias risk (77%), potentially due to inadequate EPV ratios, missing data, and unreported model calibration.^[6]^ ML-based predictive models, which employ extensive datasets, numerous variables, and diverse data types, still faced challenges, as evidenced by 32% of the models having too many predictors for the number of events (EVP < 10). This mismatch could lead to overfitting and unreliable predictions when applied to new datasets. Additionally, issues such as data collection errors, incorrect sample labeling, and external interference can compromise study integrity.^[59,60]^ Removing missing data without addressing the underlying noise can further pollute the training data, which is detrimental since supervised ML models require large, clean datasets to avoid memorizing noise and compromising model performance. To address noise in training data, researchers have suggested using robust classifiers or reverse engineering the noise generation process to correlate clean and noisy labels, thereby improving label quality.^[60–62]^ Despite 52% of the models reporting discrimination, many lacked calibration descriptions, which is crucial for evaluating the model’s accuracy in predicting absolute risk and assessing its fit.Notably, while internal validation was consistently well-performed, most models did not undergo external validation. Without this crucial step, the models remain untested in clinical settings, and their reliability and generalizability are uncertain, especially given the potential for performance degradation due to initial overfitting.^[63,64]^ In the specific context of image prediction models for inflammatory bowel disease and models predicting elevated intracranial pressure post-traumatic brain injury, high bias risks were again prevalent, often attributable to insufficient sample sizes, undisclosed exclusions, and lack of external validation groups.^[65]^ As for the applicability of prognostic models, the majority were deemed to have limited applicability and were prone to bias due to the absence of external validation.^[66]^

However, there are some limitations when applying PROBAST to ML models in its current form. Specifically, supervised ML-based predictive models can autonomously learn from data without explicit programming, identifying patterns directly from the dataset. These models offer flexibility in capturing associations and handling modeling complexity, whereas traditional prediction models rely on regression-based approaches that allow full model transparency.^[67]^ Consequently, PROBAST may not adequately account for biases unique to ML methods. To mitigate bias, understanding how predictions are derived is often more critical than knowing the predictions themselves in practical applications. For clinical practitioners, the adoption of interpretable ML tools during development is essential. Specifically, these tools employ rule-based modeling to aggregate multivariate statistics, generating accessible and transparent outcomes that help minimize analytical bias and noise while facilitating data sharing. For researchers, prioritizing the active development and updating of standardized reporting guidelines and risk-of-bias analysis tools for ML-based prediction models will enhance both study reporting quality and critical appraisal processes.

In future medical research, external validation and missing data processing will constitute 2 critical challenges for ensuring model generalizability and reliability. Before these models can be reliably used in clinical practice, they must undergo rigorous external validation to improve their predictive accuracy and ensure their effectiveness across different clinical settings. Multicenter collaboration represents an effective strategy, though it requires emphasis that rigorous data standardization and cross-center data isolation are imperative during external validation. A unified data collection protocol should be implemented to ensure standardization, while cross-center validation or federated learning frameworks can maintain data isolation. Additionally, reporting inter-center performance variations and conducting calibration analyses are essential to mitigate distributional disparities across sites. Missing data constitutes a ubiquitous but methodologically addressable challenge. Processing must align with training strategies, employing either federated imputation methods or center-stratified imputation, while strictly prohibiting recalculation of imputed values during testing to prevent information leakage. Ultimately, the final solution must achieve an optimal balance among model generalizability, computational efficiency, and privacy compliance.

## 5. Strengths and limitations

Our study focused on assessing the risk of bias in supervised ML research on prognostic models for patients undergoing TKA/THA. The goal was to improve clinical decision-making and patient care outcomes. We employed the PROBAST to ensure objectivity and consistency in our evaluations. However, our study had several limitations. The current search strategy risks over-reliance on umbrella terms (e.g., “ML”), potentially omitting algorithm-specific keywords and methodological descriptors (e.g., deep learning, random forest, or XGBoost). This approach may systematically exclude seminal studies, thus compromising the review’s validity. We propose expanding both conceptual and technical search criteria to ensure systematic literature coverage. The research was primarily condu0cted in China and the United States, and there was a notable lack of external validation. This limitation may restrict the generalizability of the models across different countries, underscoring the critical need for external validation. While our primary concern was bias risk, the issue of reporting transparency also requires attention. Although PROBAST provides a standardized evaluation framework, its criteria are not fully applicable to models developed through supervised ML. Future research will need to adopt more rigorous methodologies and improve reporting transparency to enhance the reliability of prognostic models in this context.

## 6. Conclusion

This review encompasses 32 studies and 25 models. According to the PROBAST assessment, the majority of these models present a high risk of bias, indicating significant room for improvement in both bias risk and applicability. Contributing factors to this high bias risk include inadequate sample sizes, incomplete data on recruitment, model overfitting, and a lack of extensive external validation. Consequently, rigorous compliance with established standards and substantial enhancements in the methodology of constructing supervised ML-based prognostic models are imperative. Such improvements are crucial for transforming algorithmic insights into practical tools that can improve patient care outcomes and reduce inefficiencies in research.

## Author contributions

**Conceptualization:** Hongxia Zhang, Lina Jiang, Chuanbo Li.

**Data curation:** Jiang Zheng.

**Formal analysis:** Hongxia Zhang, Lina Jiang.

**Funding acquisition:** Chuanbo Li.

**Investigation:** Jiang Zheng.

**Methodology:** Hongxia Zhang, Lina Jiang.

**Project administration:** Chuanbo Li.

**Resources:** Chuanbo Li.

**Software:** Hongxia Zhang, Jiang Zheng.

**Supervision:** Chuanbo Li.

**Validation:** Lina Jiang.

**Visualization:** Hongxia Zhang, Jiang Zheng.

**Writing – original draft:** Hongxia Zhang, Lina Jiang.

**Writing – review & editing:** Jiang Zheng, Chuanbo Li.

## Supplementary Material


