# Supplementary material for: Supervised machine learning-based bias risk of prognostic models for total knee or hip arthroplasty patients: A systematic review
Source: Medicine (Baltimore). 2025 Oct 17;104(42):e45230. doi: 10.1097/MD.0000000000045230 (PMC12537099; doi:10.1097/MD.0000000000045230)
Supplement: Supplementary file 1 [file medi-104-e45230-s001.docx]

**Supplementary Table 1. Search strategy**

Built until January 12, 2024

**(1)** **PUBMED**

| **Search** | **Query** |
| --- | --- |
| #1 | ((((((((((Artificial Intelligence[MeSH Terms]) OR (Learning, Machine[MeSH Terms])) OR (Deep Learning[MeSH Terms])) OR (Supervised Machine Learning[MeSH Terms])) OR (Neural Networks, Computer[MeSH Terms])) OR (Transfer Learning[MeSH Terms])) OR (Support Vector Machine[MeSH Terms])) OR (Random forest[MeSH Terms])) OR (naïve bayes[MeSH Terms])) OR (gradient boosting machines[MeSH Terms])) OR (data mining[MeSH Terms]) |
| #2 | ((((((((multilayer perceptron*[Title/Abstract]) OR (random forest*[Title/Abstract])) OR (bayes* network*[Title/Abstract])) OR (support vector machine*[Title/Abstract])) OR (nearest neighbor*[Title/Abstract])) OR (k-nearest neighbor*[Title/Abstract])) OR (elastic net [Title/Abstract])) OR (naive bayes*[Title/Abstract])) |
| #3 | #1 OR #2 |
| #4 | (((Validat*[MeSH Terms]) OR (Predict*[MeSH Terms])) OR (Rule*[MeSH Terms)) OR (Variable*[MeSH Terms]) |
| #5 | (((Model*[Title/Abstract]) OR (Scor*[Title/Abstract])) OR (Characteristic*[Title/Abstract])) OR (Finding*[Title/Abstract]) |
| #6 | #4 AND #5 |
| #7 | ((((((((Knee[MeSH Terms]) OR (Knee Joint*[MeSH Terms])) OR (Arthroplasty, Replacement, Knee[MeSH Terms])) OR (Knee Prosthesis[MeSH Terms])) OR (Hip[MeSH Terms])) OR (Hip Joint*[MeSH Terms])) OR (Arthroplasty, Replacement, Hip[MeSH Terms])) OR (Hip Prosthesis[MeSH Terms])) OR (Arthroplasty, Replacement[MeSH Terms]) |
| #8 | (((((((((((((((((((Knee[Title/Abstract]) OR (Knee Joint*[Title/Abstract])) OR (Arthroplasty, Knee Replacement[Title/Abstract])) OR (Knee Replacement Arthroplast*[Title/Abstract])) OR (Replacement Arthroplasties, Knee[Title/Abstract])) OR (Knee Arthroplasty, Total[Title/Abstract])) OR (Arthroplasty, Total Knee[Title/Abstract])) OR (Total Knee Arthroplasty[Title/Abstract])) OR (Replacement, Total Knee[Title/Abstract])) OR (Knee Replacement[Title/Abstract])) OR (Hip[Title/Abstract])) OR (Hip Joint*[Title/Abstract])) OR (Arthroplasty, Hip Replacement[Title/Abstract])) OR (Hip Replacement Arthroplast*[Title/Abstract])) OR (Replacement Arthroplasties, Hip[Title/Abstract])) OR (Hip Arthroplasty, Total[Title/Abstract])) OR (Arthroplasty, Total Hip[Title/Abstract])) OR (Total Hip Arthroplasty[Title/Abstract])) OR (Replacement, Total Hip[Title/Abstract])) OR (Hip Replacement[Title/Abstract]) |
| #9 | #7 OR #8 |
| #10 | #3 AND #6 AND #9 |

**(2) Web of Science**

| **Search** | **Query** |
| --- | --- |
| #1 | TS=(“Artificial Intelligence” OR “Learning, Machine” OR “Deep Learning” OR “Supervised Machine Learning” OR “Neural Networks, Computer” OR “Transfer Learning” OR “Support Vector Machine” OR “Random forest” OR “naïve bayes” OR “gradient boosting machines” OR “data mining” OR “k-nearest neighbor” OR “multilayer perceptron” OR “elastic net”) |
| #2 | TS=(“Validat*” OR “Predict*” OR “Rule*” OR “Variable*”) |
| #3 | TS=(“Model*” OR “Scor*” OR “Characteristic*” OR “Finding*”) |
| #4 | TS=(“Knee” OR “Knee Joint“ OR “Arthroplasty, Knee Replacement” OR “Knee Replacement Arthroplast*” OR “Replacement Arthroplasties, Knee” OR “Knee Arthroplasty, Total” OR “Arthroplasty, Total Knee” OR “Total Knee Arthroplasty” OR “Replacement, Total Knee” OR “Knee Replacement” OR “Hip” OR “Hip Joint*” OR “Arthroplasty, Hip Replacement” OR “Hip Replacement Arthroplast*” OR “Replacement Arthroplasties, Hip” OR “Hip Arthroplasty, Total” OR “Arthroplasty, Total Hip” OR “Total Hip Arthroplasty” OR “Replacement, Total Hip” OR “Hip Replacement”) |
| #5 | #1 AND #2 AND #3 AND #4 |

**(3) Cochrane library**

| **Search** | **Query** |
| --- | --- |
| #1 | MeSH descriptor: [Artificial Intelligence] explode all trees |
| #2 | MeSH descriptor: [Machine Learning] explode all trees |
| #3 | MeSH descriptor: [Deep Learning] explode all trees |
| #4 | MeSH descriptor: [Supervised Machine Learning] explode all trees |
| #5 | MeSH descriptor: [Neural Networks, Computer] explode all trees |
| #6 | MeSH descriptor: [Transfer, Psychology] explode all trees |
| #7 | MeSH descriptor: [Random Forest] explode all trees |
| #8 | MeSH descriptor: [Data Mining] explode all trees |
| #9 | #1 OR #2 OR #3 OR #4 OR #5 OR #6 OR #7 OR #8 |
| #10 | (“multilayer perceptron” OR “random forest” OR “bayes network” OR “support vector machine” OR “nearest neighbor” OR “k-nearest neighbor” OR “elastic net” OR “naive bayes” OR “data mining” OR “Learning, Machine” OR “Deep Learning” OR “Supervised Machine Learning” OR “Neural Networks, Computer” OR “Transfer Learning”):ti,ab,kw |
| #11 | #9 OR #10 |
| #12 | (“Validate” OR “Validation” OR “Predicte” OR “Prediction” OR “Rule” OR “Variable”):ti,ab,kw |
| #13 | (“Model” OR “Score” OR “Characteristic” OR “Finding”):ti,ab,kw |
| #14 | #12 AND #13 |
| #15 | MeSH descriptor: [Knee] explode all trees |
| #16 | MeSH descriptor: [Knee Joint] explode all trees |
| #17 | MeSH descriptor: [Arthroplasty, Replacement, Knee] explode all trees |
| #18 | MeSH descriptor: [Hip] explode all trees |
| #19 | MeSH descriptor: [Hip Joint] explode all trees |
| #20 | MeSH descriptor: [Arthroplasty, Replacement, Hip] explode all trees |
| #21 | #15 OR #16 OR #17 OR #18 OR #19 OR #20 |
| #22 | (“Knee” OR “Knee Joint“ OR “Arthroplasty, Knee Replacement” OR “Knee Replacement Arthroplast” OR “Replacement Arthroplasties, Knee” OR “Knee Arthroplasty, Total” OR “Arthroplasty, Total Knee” OR “Total Knee Arthroplasty” OR “Replacement, Total Knee” OR “Knee Replacement” OR “Hip” OR “Hip Joint” OR “Arthroplasty, Hip Replacement” OR “Hip Replacement Arthroplast” OR “Replacement Arthroplasties, Hip” OR “Hip Arthroplasty, Total” OR “Arthroplasty, Total Hip” OR “Total Hip Arthroplasty” OR “Replacement, Total Hip” OR “Hip Replacement”):ti,ab,kw |
| #23 | #21 OR #22 |
| #24 | #11 AND #14 AND #23 |

**(4) EMBASE**

| **Search** | **Query** |
| --- | --- |
| #1 | 'artificial intelligence'/exp OR 'machine learning'/exp OR 'deep learning'/exp OR 'supervised machine learning'/exp OR 'artificial neural network'/exp OR 'transfer of learning'/exp OR 'support vector machine'/exp OR 'random forest'/exp OR 'data mining'/exp |
| #2 | 'artificial intelligence':ab,ti OR 'machine learning':ab,ti OR 'deep learning':ab,ti OR 'supervised machine learning':ab,ti OR 'artificial neural network':ab,ti OR 'transfer of learning':ab,ti OR 'support vector machine':ab,ti OR 'random forest':ab,ti OR 'data mining':ab,ti OR 'multilayer perceptron':ab,ti OR 'bayesian network':ab,ti OR 'nearest neighbor algorithm':ab,ti OR 'k nearest neighbor':ab,ti OR 'elastic tissue':ab,ti OR 'bayesian learning':ab,ti |
| #3 | #1 OR #2 |
| #4 | 'validation process'/exp OR 'validation study'/exp OR ('prediction'/exp AND 'forecasting'/exp) OR 'prediction'/exp OR 'heuristics'/exp |
| #5 | 'validation process':ab,ti OR medicine:ab,ti OR (prediction:ab,ti AND forecasting:ab,ti) OR prediction:ab,ti OR heuristics:ab,ti |
| #6 | #4 OR #5 |
| #7 | 'model'/exp OR 'score'/exp |
| #8 | 'model':ab,ti OR 'score':ab,ti |
| #9 | #7 OR #8 |
| #10 | #6 AND #9 |
| #11 | 'knee disease'/exp OR 'knee osteoarthritis'/exp OR 'knee replacement'/exp OR 'knee prosthesis'/exp OR 'hip'/exp OR 'hip disease'/exp OR 'hip replacement'/exp OR 'hip prosthesis'/exp OR 'replacement arthroplasty'/exp |
| #12 | 'knee disease':ab,ti OR 'knee joint':ab,ti OR 'knee osteoarthritis':ab,ti OR 'knee replacement':ab,ti OR 'knee injury':ab,ti OR 'knee prosthesis':ab,ti OR 'total knee arthroplasty':ab,ti OR hip:ab,ti OR 'medical ethics':ab,ti OR 'hip disease':ab,ti OR 'hip prosthesis':ab,ti OR 'hip arthroplasty':ab,ti OR 'hip replacement':ab,ti OR 'hip osteoarthritis':ab,ti OR 'total hip replacement':ab,ti |
| #13 | #11 OR #12 |
| #14 | #3 AND #10 AND #13 |

**（5）CNKI**

“人工智能 OR 机器算法 OR 监督机器学习 OR 深度学习 OR 神经网络 OR 支持向量机 OR 随机森林 OR 贝叶斯网络 OR 梯度提升器 OR 数据挖掘” AND “预测 OR 验证” AND “模型 OR 风险分数 OR 列线图” AND “膝关节置换 OR 全膝关节置换 OR 髋关节置换 OR 全髋关节置换 OR 关节置换 OR TKA OR THA”

**（6）WanFang**

主题:(人工智能 OR 机器算法 OR 监督机器学习 OR 深度学习 OR 神经网络 OR 支持向量机 OR 随机森林 OR 贝叶斯网络 OR 梯度提升器 OR 数据挖掘) and 主题:(预测 OR 验证) and 主题:(模型 OR 风险分数 OR 列线图) and 主题:(膝关节置换 OR 全膝关节置换 OR 髋关节置换 OR 全髋关节置换 OR 关节置换 OR TKA OR THA)

**1. Risk of bias**

| **Author (year)** | **1 Participants** | | | **2 Predictors** | | | | **3 Outcomes** | | | | | | | **4 Analysis** | | | | | | | | | **overall risk** |
| --- | --- | --- | --- | --- | --- | --- | --- | --- | --- | --- | --- | --- | --- | --- | --- | --- | --- | --- | --- | --- | --- | --- | --- | --- |
|  | **1.1** | **1.2** | **risk** | **2.1** | **2.2** | **2.3** | **risk** | **3.1** | **3.2** | **3.3** | **3.4** | **3.5** | **3.6** | **risk** | **4.1** | **4.2** | **4.3** | **4.4** | **4.5** | **4.6** | **4.7** | **4.8** | **risk** |  |
| **Development** | | | | | | | | | | | | | | | | | | | | | | | | |
| Lungu (2015) | + | + | L | + | + | + | L | + | + | - | + | + | + | H | - | + | + | - | ? | + | - | + | H | **H** |
| Gabriel (2019) | - | + | H | + | + | + | L | + | + | + | - | ? | + | H | ? | ? | - | - | + | + | + | + | H | **H** |
| Katakam (2020) | + | + | H | + | + | + | L | - | + | + | - | ? | + | L | + | ? | - | - | + | + | + | + | H | **H** |
| Chen (2021) | - | + | H | + | + | + | L | + | ? | + | - | + | + | H | - | ? | + | + | + | + | - | + | H | **H** |
| Chen (2021) | - | + | H | - | + | + | H | + | + | + | + | + | + | L | - | - | + | + | - | ? | - | + | H | **H** |
| Fassihi (2021) | + | + | L | - | ? | + | H | + | ? | ? | + | ? | + | UN | ? | + | + | + | - | - | - | + | H | **H** |
| Han (2021) | - | + | H | + | + | + | H | + | ? | ? | + | ? | + | UN | + | + | + | + | + | + | + | + | L | **H** |
| Huang (2021) | + | + | L | + | + | + | L | - | ? | ? | + | ? | + | H | + | ? | - | - | ? | + | + | + | H | **H** |
| Jamshidi (2021) | + | + | L | + | + | + | L | ? | ? | ? | + | + | + | UN | + | - | + | + | + | + | - | + | H | **H** |
| Wei (2021) | + | + | L | + | + | + | L | + | + | + | + | + | + | L | + | ? | + | + | + | + | - | + | H | **H** |
| Yeo (2021) | + | + | L | + | + | + | L | + | + | + | + | + | ? | UN | + | ? | + | + | + | + | - | + | H | **H** |
| Zhang (2021) | + | + | L | + | + | + | L | + | + | + | + | + | + | L | + | ? | - | + | + | + | + | + | H | **H** |
| Zhong (2021) | - | + | H | + | + | + | L | - | + | + | - | + | + | L | + | + | + | + | + | + | + | + | L | **H** |
| Chen (2022) | + | + | L | + | + | + | L | + | + | + | + | + | + | L | - | ? | + | + | + | ? | + | + | H | **H** |
| Cohen‑Levy (2022) | - | + | H | + | + | + | L | ? | + | + | + | + | ? | UN | + | ? | + | ? | + | + | + | + | UN | **H** |
| Dezfouli (2022) | + | - | H | + | + | + | L | + | + | + | + | + | ? | H | + | ? | - | ? | ? | + | + | + | H | **H** |
| Kunze (2022) | + | + | L | + | + | + | L | + | + | + | + | + | + | L | + | ? | ? | ? | + | ? | + | + | UN | **UN** |
| Lopez (2022) | + | + | L | + | + | + | L | + | + | + | + | + | + | L | + | - | - | + | + | + | + | + | H | **H** |
| Mohammed (2022) | - | + | H | + | - | ? | H | + | + | ? | + | + | ? | UN | + | - | - | + | ? | + | + | + | H | **H** |
| Zheng (2022) | - | + | H | - | + | + | H | + | + | + | + | + | + | L | - | + | + | + | + | + | - | + | H | **H** |
| Cavazos (2023) | - | ? | H | + | + | + | L | ? | + | + | + | + | ? | UN | + | + | + | + | + | + | + | + | L | **H** |
| Chen (2023) | - | + | H | + | + | - | H | + | + | + | + | + | ? | UN | + | ? | + | ? | ? | + | + | + | UN | **H** |
| Crawford (2023) | - | + | H | + | + | + | L | ? | ? | ? | + | + | ? | UN | - | ? | - | + | + | + | + | + | H | **H** |
| Ding (2023) | - | + | H | + | + | + | L | + | + | + | + | + | ? | UN | - | + | - | + | + | + | + | + | H | **H** |
| Jia (2023) | - | + | H | + | + | - | H | + | + | + | + | + | + | L | + | ? | ? | ? | ? | + | - | + | H | **H** |
| Klemt (2023) | + | + | L | + | + | + | L | + | + | + | + | + | + | L | + | - | - | + | + | + | + | + | H | **H** |
| Nam (2023) | + | + | L | + | + | + | L | + | + | + | + | + | + | L | - | ? | + | + | + | + | - | + | H | **H** |
| Park (2023) | + | + | L | + | + | + | L | + | + | + | + | + | + | L | - | + | - | - | + | + | - | + | H | **H** |
| Wang (2023) | + | + | L | + | + | + | L | + | + | + | + | + | + | L | + | + | + | + | + | + | - | + | L | **H** |
| Yeramosu (2023) | + | + | L | + | + | + | L | ? | ? | ? | + | ? | ? | UN | + | ? | + | + | + | + | - | + | L | **H** |
| **Validation** | | | | | | | | | | | | | | | | | | | | | | | | |
| Twiggs (2019) | + | ? | UN | - | + | + | H | + | + | - | - | + | + | H | - | + | ? | ? | ? | ? | - | ? | H | **H** |
| **Development and Validation** | | | | | | | | | | | | | | | | | | | | | | | | |
| Chen (2023) | - | + | H | + | + | + | L | + | + | + | + | + | ? | UN | + | ? | ? | ? |  | + | + |  | UN | **H** |

+, Yes/Probably Yes; -, Not/Possibly not; ?, No Information; L, Low; H ,High; UN, Unclear.

**2. Applicability**

| **Author (year)** | **Participants** | **Predictors** | **Outcomes** | Overall applicability |
| --- | --- | --- | --- | --- |
| **Development** | | | | |
| Lungu (2015) | + | + | + | L |
| Gabriel (2019) | - | + | - | H |
| Katakam (2020) | + | + | - | H |
| Chen (2021) | - | + | - | H |
| Chen (2021) | - | - | + | H |
| Fassihi (2021) | + | - | ? | H |
| Han (2021) | - | + | ? | H |
| Huang (2021) | + | + | ? | UN |
| Jamshidi (2021) | + | + | ? | UN |
| Wei (2021) | + | + | + | L |
| Yeo (2021) | + | + | + | L |
| Zhang (2021) | + | + | + | L |
| Zhong (2021) | - | + | - | H |
| Chen (2022) | + | + | + | L |
| Cohen‑Levy (2022) | - | + | ? | H |
| Dezfouli (2022) | + | + | ? | UN |
| Kunze (2022) | + | + | + | L |
| Lopez (2022) | + | + | + | L |
| Mohammed (2022) | - | + | ? | H |
| Zheng (2022) | - | - | + | H |
| Cavazos (2023) | - | + | ? | H |
| Chen (2023) | - | - | ? | H |
| Crawford (2023) | - | + | ? | H |
| Ding (2023) | - | + | ? | H |
| Jia (2023) | - | - | + | H |
| Klemt (2023) | + | + | + | L |
| Nam (2023) | + | + | + | L |
| Park (2023) | + | + | + | L |
| Wang (2023) | + | + | + | L |
| Yeramosu (2023) | + | + | ? | UN |
| **Validation** | | | | |
| Twiggs (2019) | + | - | - | H |
| **Development and Validation** | | | | |
| Chen (2023) | - | + | ? | H |

+, Yes/Probably Yes; -, Not/Possibly not; ?, No Information; L, Low; H ,High; UN, Unclear.
